# Supplementary material for: Creating bulk ultrastable glasses by random particle bonding
Source: Nat Commun. 2023 Jan 7;14:113. doi: 10.1038/s41467-023-35812-w (PMC9825381; doi:10.1038/s41467-023-35812-w)
Supplement: Supplementary file 1 — Supplementary Information [file 41467_2023_35812_MOESM1_ESM.pdf]

Supplementary Information:
Creating bulk ultrastable glasses by random particle bonding

Misaki Ozawa,[1] Yasutaka Iwashita,[2] Walter Kob,[3] and Francesco Zamponi[1]

[1] *Laboratoire de Physique de l'Ecole normale supérieure, ENS, Université PSL, CNRS, Sorbonne Université, Université Paris-Diderot, Sorbonne Paris Cité, Paris, France*

[2] *Department of Physics, Kyoto Sangyo University, Kyoto, Japan*

[3] *Laboratoire Charles Coulomb, University of Montpellier and CNRS, F-34095 Montpellier,France*

Supplementary Note 1. STATISTICAL MECHANICS OF BONDED SYSTEMS

We discuss here the statistical mechanics of randomly-bonded glass formers composed of monomers and dimers. For this we randomly choose a fraction of pairs of neighboring particles from an equilibrium configuration and create bonds with a rigid-body constraint. This section also defines some of the notation used in the main text of the manuscript.

A. Variable transformation

Consider a system of N point particles in three dimensions, $d = 3$, such as the Kob-Andersen model [1]. The Hamiltonian H and the partition function Z are given by

$$H(\mathbf{r}^N, \mathbf{p}^N) = \sum_{i=1}^{N} \frac{\mathbf{p}_i^2}{2m_i} + U(\mathbf{r}^N) \ , \qquad Z = \int \mathrm{d}\mathbf{r}^N \mathrm{d}\mathbf{p}^N \ \exp[-\beta H(\mathbf{r}^N, \mathbf{p}^N)] \ , \tag{1}$$

where $\mathbf{r}_i$, $\mathbf{p}_i$, and m_i are the position, momentum, and mass of the i-th particle, respectively. U is the potential energy, and $\beta = 1/T$ is the inverse temperature. In this paper, we use a shorthand notation for a vector of N variables, e.g., $\mathbf{r}^N = (\mathbf{r}_1, \mathbf{r}_2, ..., \mathbf{r}_N)$. Note that we omit the combinatorial factors such as $N!$ and the Planck's constant h, because we will not discuss the absolute value of the free energy or entropy in this work.

We consider the following variable transformation from Cartesian coordinates to Jacobi coordinates. We assign virtual bonds between two neighboring particles chosen randomly from an equilibrium configuration, as schematically shown in Supplementary Figure S1a. Note that this is a virtual operation, and the actual system is not altered at all, i.e., this is merely a variable transformation. After the transformation, N particles are classified into the group of N_m monomers and the others into the group of N_d dimers. By construction, $N = N_\mathrm{m} + 2N_\mathrm{d}$. The index of the particle associated with the monomers and dimers belongs to sets $i \in \mathcal{M}$ and $i \in \mathcal{D}$, respectively.

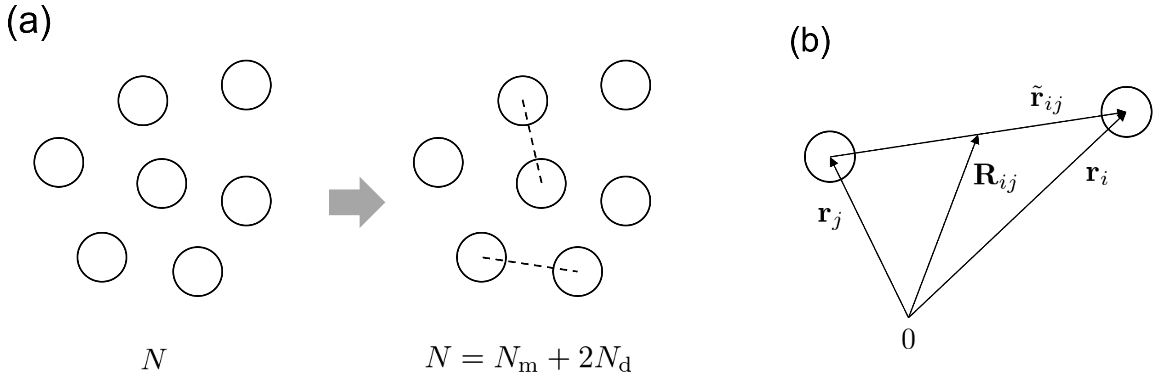

Supplementary Figure S1. **Schematic plots for statistical mechanics of bonded systems.** (a): Making virtual bonds. (b): The center of mass and relative position describe a dimer connecting particles i and j.

For the monomers, we continue to use the Cartesian coordinates. For the dimers, instead, we use the Jacobi coordinates for the two-body problem, using the center of mass of two particles and relative position as shown in

Supplementary Figure S1b. The center of mass and relative position for a dimer composed of the i-th and j-th particles are given by $\mathbf{R}_{ij} = \frac{m_i\mathbf{r}_i+m_j\mathbf{r}_j}{m_i+m_j}$ and $\tilde{\mathbf{r}}_{ij} = \mathbf{r}_i - \mathbf{r}_j$, respectively. The corresponding momenta are denoted by $\mathbf{P}_{ij}$ and $\tilde{\mathbf{p}}_{ij}$, respectively. Also, the total mass and the reduced mass are given by $M_{ij} = m_i + m_j$ and $\mu_{ij} = \frac{m_i m_j}{m_i+m_j}$, respectively. The described variable transformation is formally written by $(\mathbf{r}^N, \mathbf{p}^N) \to (\mathbf{r}^{N_\mathrm{m}}, \mathbf{p}^{N_\mathrm{m}}, \mathbf{R}^{N_\mathrm{d}}, \mathbf{P}^{N_\mathrm{d}}, \tilde{\mathbf{r}}^{N_\mathrm{d}}, \tilde{\mathbf{p}}^{N_\mathrm{d}})$.

Under this variable transformation, we can rewrite the Hamiltonian H as,

$$H(\mathbf{r}^{N_\mathrm{m}}, \mathbf{p}^{N_\mathrm{m}}, \mathbf{R}^{N_\mathrm{d}}, \mathbf{P}^{N_\mathrm{d}}, \tilde{\mathbf{r}}^{N_\mathrm{d}}, \tilde{\mathbf{p}}^{N_\mathrm{d}}) = \sum_{i\in\mathcal{M}} \frac{\mathbf{p}_i^2}{2m_i} + \sum_{\substack{i,j\in\mathcal{D}\\(i<j)}} \left(\frac{\mathbf{P}_{ij}^2}{2M_{ij}} + \frac{\tilde{\mathbf{p}}_{ij}^2}{2\mu_{ij}} \right) + U(\mathbf{r}^{N_\mathrm{m}}, \mathbf{R}^{N_\mathrm{d}}, \tilde{\mathbf{r}}^{N_\mathrm{d}}) \ , \tag{2}$$

and the partition function Z as

$$Z = \int (\prod_{i\in\mathcal{M}} \mathrm{d}\mathbf{r}_i \mathrm{d}\mathbf{p}_i) \int (\prod_{\substack{i,j\in\mathcal{D}\\(i<j)}} \mathrm{d}\mathbf{R}_{ij} \mathrm{d}\mathbf{P}_{ij}) \int (\prod_{\substack{i,j\in\mathcal{D}\\(i<j)}} \mathrm{d}\tilde{\mathbf{r}}_{ij} \mathrm{d}\tilde{\mathbf{p}}_{ij}) \exp[-\beta H(\mathbf{r}^{N_\mathrm{m}}, \mathbf{p}^{N_\mathrm{m}}, \mathbf{R}^{N_\mathrm{d}}, \mathbf{P}^{N_\mathrm{d}}, \tilde{\mathbf{r}}^{N_\mathrm{d}}, \tilde{\mathbf{p}}^{N_\mathrm{d}})] \ . \tag{3}$$

We further proceed with the variable transformation for the relative movements of dimers by using spherical coordinates in $d = 3$, which is formally written as $(\tilde{\mathbf{r}}^{N_\mathrm{d}}, \tilde{\mathbf{p}}^{N_\mathrm{d}}) \to (\tilde{r}^{N_\mathrm{d}}, \theta^{N_\mathrm{d}}, \varphi^{N_\mathrm{d}}, p_{\tilde{r}}^{N_\mathrm{d}}, p_\theta^{N_\mathrm{d}}, p_\varphi^{N_\mathrm{d}})$. The Liouville's theorem ensures that the Jacobian is unity for this transformation, namely, $\mathrm{d}\tilde{\mathbf{r}}_{ij}\mathrm{d}\tilde{\mathbf{p}}_{ij} = \mathrm{d}\tilde{r}_{ij}\mathrm{d}\theta_{ij}\mathrm{d}\varphi_{ij}\mathrm{d}p_{\tilde{r}_{ij}}\mathrm{d}p_{\theta_{ij}}\mathrm{d}p_{\varphi_{ij}}$. Also, the kinetic part of the Hamiltonian can be written as

$$\frac{\tilde{\mathbf{p}}_{ij}^2}{2\mu_{ij}} = \frac{p_{\tilde{r}_{ij}}^2}{2\mu_{ij}} + \frac{p_{\theta_{ij}}^2}{2I_{ij}} + \frac{p_{\varphi_{ij}}^2}{2I_{ij}\sin^2\theta_{ij}} \ , \tag{4}$$

where $I_{ij} = \mu_{ij}\tilde{r}_{ij}^2$ is the moment of inertia, $p_{\tilde{r}_{ij}} = \mu_{ij}\dot{\tilde{r}}_{ij}$, $p_{\theta_{ij}} = I_{ij}\dot{\theta}_{ij}$, and $p_{\varphi_{ij}} = I_{ij}(\sin^2\theta_{ij})\dot{\varphi}_{ij}$ are new momenta. We note that as one can see in Eq. (4), the kinetic term also depends on the coordinates. Thus we cannot treat the momenta and positions separately, unlike in the random pinning setting.

We finally arrive at the expressions for the Hamiltonian H, the partition function Z and the Boltzmann distribution $\rho = \exp[-\beta H]/Z$ that are suitable for our study:

$$\begin{aligned} &H(\mathbf{r}^{N_\mathrm{m}}, \mathbf{p}^{N_\mathrm{m}}, \mathbf{R}^{N_\mathrm{d}}, \mathbf{P}^{N_\mathrm{d}}, \tilde{r}^{N_\mathrm{d}}, \theta^{N_\mathrm{d}}, \varphi^{N_\mathrm{d}}, p_{\tilde{r}}^{N_\mathrm{d}}, p_\theta^{N_\mathrm{d}}, p_\varphi^{N_\mathrm{d}}) = \\ &\sum_{i\in\mathcal{M}} \frac{\mathbf{p}_i^2}{2m_i} + \sum_{\substack{i,j\in\mathcal{D}\\(i<j)}} \left(\frac{\mathbf{P}_{ij}^2}{2M_{ij}} + \frac{p_{\tilde{r}_{ij}}^2}{2\mu_{ij}} + \frac{p_{\theta_{ij}}^2}{2I_{ij}} + \frac{p_{\varphi_{ij}}^2}{2I_{ij}\sin^2\theta_{ij}} \right) + U(\mathbf{r}^{N_\mathrm{m}}, \mathbf{R}^{N_\mathrm{d}}, \tilde{r}^{N_\mathrm{d}}, \theta^{N_\mathrm{d}}, \varphi^{N_\mathrm{d}}) \ , \end{aligned} \tag{5}$$

$$\begin{aligned} Z = &\int (\prod_{i\in\mathcal{M}} \mathrm{d}\mathbf{r}_i \mathrm{d}\mathbf{p}_i) \int (\prod_{\substack{i,j\in\mathcal{D}\\(i<j)}} \mathrm{d}\mathbf{R}_{ij} \mathrm{d}\mathbf{P}_{ij}) \int (\prod_{\substack{i,j\in\mathcal{D}\\(i<j)}} \mathrm{d}\tilde{r}_{ij}\mathrm{d}\theta_{ij}\mathrm{d}\varphi_{ij}\mathrm{d}p_{\tilde{r}_{ij}}\mathrm{d}p_{\theta_{ij}}\mathrm{d}p_{\varphi_{ij}}) \\ &\times \exp[-\beta H(\mathbf{r}^{N_\mathrm{m}}, \mathbf{p}^{N_\mathrm{m}}, \mathbf{R}^{N_\mathrm{d}}, \mathbf{P}^{N_\mathrm{d}}, \tilde{r}^{N_\mathrm{d}}, \theta^{N_\mathrm{d}}, \varphi^{N_\mathrm{d}}, p_{\tilde{r}}^{N_\mathrm{d}}, p_\theta^{N_\mathrm{d}}, p_\varphi^{N_\mathrm{d}})] \ . \end{aligned} \tag{6}$$

We define the average over the total Boltzmann distribution, $\langle\cdots\rangle$, by

$$\begin{aligned} \langle\cdots\rangle = &\int (\prod_{i\in\mathcal{M}} \mathrm{d}\mathbf{r}_i \mathrm{d}\mathbf{p}_i) \int (\prod_{\substack{i,j\in\mathcal{D}\\(i<j)}} \mathrm{d}\mathbf{R}_{ij} \mathrm{d}\mathbf{P}_{ij}) \int (\prod_{\substack{i,j\in\mathcal{D}\\(i<j)}} \mathrm{d}\tilde{r}_{ij}\mathrm{d}\theta_{ij}\mathrm{d}\varphi_{ij}\mathrm{d}p_{\tilde{r}_{ij}}\mathrm{d}p_{\theta_{ij}}\mathrm{d}p_{\varphi_{ij}}) \\ &\times \rho(\mathbf{r}^{N_\mathrm{m}}, \mathbf{p}^{N_\mathrm{m}}, \mathbf{R}^{N_\mathrm{d}}, \mathbf{P}^{N_\mathrm{d}}, \tilde{r}^{N_\mathrm{d}}, \theta^{N_\mathrm{d}}, \varphi^{N_\mathrm{d}}, p_{\tilde{r}}^{N_\mathrm{d}}, p_\theta^{N_\mathrm{d}}, p_\varphi^{N_\mathrm{d}})(\cdots). \end{aligned} \tag{7}$$

We note again that up to this point, we have just discussed an exact variable transformation. We did not modify the system itself at all.

B. Bonded systems

Using the notations introduced above, we now consider bonded systems. We freeze $\tilde{r}^{N_\mathrm{d}}$ from an equilibrium configuration, i.e., $\tilde{r}^{N_\mathrm{d}}$ plays the same role as the positions of the pinned particles in the random pinning approach.

Thus we now can discuss the statistical mechanics of the remaining degrees of freedom, namely a mixture of monomers and dimers for a particular realization of $\tilde{r}^{N_\mathrm{d}}$. The Hamiltonian of this setting, H', is given by

$$\begin{aligned}
&H'(\mathbf{r}^{N_\mathrm{m}}, \mathbf{p}^{N_\mathrm{m}}, \mathbf{R}^{N_\mathrm{d}}, \mathbf{P}^{N_\mathrm{d}}, \tilde{r}^{N_\mathrm{d}}, \theta^{N_\mathrm{d}}, \varphi^{N_\mathrm{d}}, p_\theta^{N_\mathrm{d}}, p_\varphi^{N_\mathrm{d}}) = \\
&\quad \sum_{i\in\mathcal{M}} \frac{\mathbf{p}_i^2}{2m_i} + \sum_{\substack{i,j\in\mathcal{D}\\(i<j)}} \left(\frac{\mathbf{P}_{ij}^2}{2M_{ij}} + \frac{p_{\theta_{ij}}^2}{2I_{ij}} + \frac{p_{\varphi_{ij}}^2}{2I_{ij}\sin^2\theta_{ij}} \right) + U(\mathbf{r}^{N_\mathrm{m}}, \mathbf{R}^{N_\mathrm{d}}, \tilde{r}^{N_\mathrm{d}}, \theta^{N_\mathrm{d}}, \varphi^{N_\mathrm{d}}) \\
&\quad = H(\mathbf{r}^{N_\mathrm{m}}, \mathbf{p}^{N_\mathrm{m}}, \mathbf{R}^{N_\mathrm{d}}, \mathbf{P}^{N_\mathrm{d}}, \tilde{r}^{N_\mathrm{d}}, \theta^{N_\mathrm{d}}, \varphi^{N_\mathrm{d}}, p_{\tilde{r}}^{N_\mathrm{d}}, p_\theta^{N_\mathrm{d}}, p_\varphi^{N_\mathrm{d}}) - \sum_{\substack{i,j\in\mathcal{D}\\(i<j)}} \frac{p_{\tilde{r}_{ij}}^2}{2\mu_{ij}}.
\end{aligned} \tag{8}$$

The partition function Z' for a given realization $\tilde{r}^{N_\mathrm{d}}$, is

$$\begin{aligned}
Z'(\tilde{r}^{N_\mathrm{d}}) = &\int (\prod_{i\in\mathcal{M}} \mathrm{d}\mathbf{r}_i \mathrm{d}\mathbf{p}_i) \int (\prod_{\substack{i,j\in\mathcal{D}\\(i<j)}} \mathrm{d}\mathbf{R}_{ij}\mathrm{d}\mathbf{P}_{ij}) \int (\prod_{\substack{i,j\in\mathcal{D}\\(i<j)}} \mathrm{d}\theta_{ij}\mathrm{d}\varphi_{ij}\mathrm{d}p_{\theta_{ij}}\mathrm{d}p_{\varphi_{ij}}) \\
&\times \exp[-\beta H'(\mathbf{r}^{N_\mathrm{m}}, \mathbf{p}^{N_\mathrm{m}}, \mathbf{R}^{N_\mathrm{d}}, \mathbf{P}^{N_\mathrm{d}}, \tilde{r}^{N_\mathrm{d}}, \theta^{N_\mathrm{d}}, \varphi^{N_\mathrm{d}}, p_\theta^{N_\mathrm{d}}, p_\varphi^{N_\mathrm{d}})].
\end{aligned} \tag{9}$$

The corresponding conditional probability distribution given $\tilde{r}^{N_\mathrm{d}}$ is written by

$$\begin{aligned}
&\rho(\mathbf{r}^{N_\mathrm{m}}, \mathbf{p}^{N_\mathrm{m}}, \mathbf{R}^{N_\mathrm{d}}, \mathbf{P}^{N_\mathrm{d}}, \theta^{N_\mathrm{d}}, \varphi^{N_\mathrm{d}}, p_\theta^{N_\mathrm{d}}, p_\varphi^{N_\mathrm{d}} \mid \tilde{r}^{N_\mathrm{d}}) = \\
&\quad \frac{1}{Z'(\tilde{r}^{N_\mathrm{d}})} \exp[-\beta H'(\mathbf{r}^{N_\mathrm{m}}, \mathbf{p}^{N_\mathrm{m}}, \mathbf{R}^{N_\mathrm{d}}, \mathbf{P}^{N_\mathrm{d}}, \tilde{r}^{N_\mathrm{d}}, \theta^{N_\mathrm{d}}, \varphi^{N_\mathrm{d}}, p_\theta^{N_\mathrm{d}}, p_\varphi^{N_\mathrm{d}})].
\end{aligned} \tag{10}$$

Hence a thermal average for a particular realization $\tilde{r}^{N_\mathrm{d}}$ is defined by

$$\begin{aligned}
\langle \cdots \rangle_{\tilde{r}^{N_\mathrm{d}}} = &\int (\prod_{i\in\mathcal{M}} \mathrm{d}\mathbf{r}_i \mathrm{d}\mathbf{p}_i) \int (\prod_{\substack{i,j\in\mathcal{D}\\(i<j)}} \mathrm{d}\mathbf{R}_{ij}\mathrm{d}\mathbf{P}_{ij}) \int (\prod_{\substack{i,j\in\mathcal{D}\\(i<j)}} \mathrm{d}\theta_{ij}\mathrm{d}\varphi_{ij}\mathrm{d}p_{\theta_{ij}}\mathrm{d}p_{\varphi_{ij}}) \\
&\times \rho(\mathbf{r}^{N_\mathrm{m}}, \mathbf{p}^{N_\mathrm{m}}, \mathbf{R}^{N_\mathrm{d}}, \mathbf{P}^{N_\mathrm{d}}, \theta^{N_\mathrm{d}}, \varphi^{N_\mathrm{d}}, p_\theta^{N_\mathrm{d}}, p_\varphi^{N_\mathrm{d}} \mid \tilde{r}^{N_\mathrm{d}})(\cdots).
\end{aligned} \tag{11}$$

Because the choice of the bonds $\tilde{r}^{N_\mathrm{d}}$ that are frozen depends on the other degrees of freedom (i.e., only neighboring particles are chosen to be bonded), we cannot use a simple probability chain rule to relate the thermal average at fixed $\tilde{r}^{N_\mathrm{d}}$ with the total thermal average as done in random pinning [2]. We will discuss this point in more detail in a future publication.

Supplementary Note 2. SIMULATION METHODS

A. Model

We employ the Kob-Andersen binary mixture [1], in which particles interact through the Lennard-Jones potential,

$$u_{\alpha\beta}(r) = 4\epsilon_{\alpha\beta}\left[\left(\frac{\sigma_{\alpha\beta}}{r}\right)^{12} - \left(\frac{\sigma_{\alpha\beta}}{r}\right)^{6}\right], \tag{12}$$

where $\alpha, \beta = A, B$ are species indexes. Both species have the same mass, which is set to $m = 1$. The value of the parameters $\sigma_{\alpha\beta}$ and $\epsilon_{\alpha\beta}$ are given in Ref. [1]. The units of length and energy are set by the parameters $\sigma = \sigma_{\mathrm{AA}} = 1$ and $\epsilon = \epsilon_{\mathrm{AA}} = 1$, respectively, and we set the Boltzmann constant $k_\mathrm{B} = 1$. The potentials are cut and shifted at a distance $2.5\sigma_{\alpha\beta}$. We simulate systems composed of N particles in a cubic box of side L with periodic boundary conditions at a number density $\rho = N/V = 1.2$. We use the system size $N = 1200$ for the study of equilibrium dynamics and $N = 32400$ for the non-equilibrium heating-cooling process and athermal mechanical test. We use the LAMMPS simulation package [3] to generate equilibrium configurations of the original KA model.

B. Making randomly bonded systems

Starting from an equilibrium configuration of the original KA model with N particles described above, we generate a randomly bonded system composed of monomers and dimers. The algorithm to do so is as follows. First, we choose a particle randomly, say particle i. We then choose another particle j randomly among the neighboring particles of particle i, located inside a sphere with the radius R_b and which is not yet bonded, as schematically shown in Supplementary Figure S2a. We set $R_\mathrm{b} = 1.5$, which is near the first minimum of the radial distribution function, thus corresponding roughly to the boundary of the first coordination shell. We then freeze the distance between the two particles, $\tilde{r}_{ij} = |\mathbf{r}_i - \mathbf{r}_j|$, permanently, which means that the particles i and j now form a dimer. We repeat the above process for the remaining monomer particles until the number of dimers, N_d, reaches the target value. By construction, we have $N = N_\mathrm{m} + 2N_\mathrm{d}$, where N_m is the number of monomers. We introduce the control parameter $c = \frac{2N_\mathrm{d}}{N} = \frac{N-N_\mathrm{m}}{N}$, such that $c = 0$ corresponds to the system with only monomers (hence the original non-bonded system), whereas $c = 1$ corresponds to a system having only dimers. Using the algorithm explained above, it is difficult in practice to reach $c = 1$, because at some point we run out of neighboring pairs, leaving a few percent of monomer particles. Thus we use $c = 0.95$ as the maximum value that we study in this work.

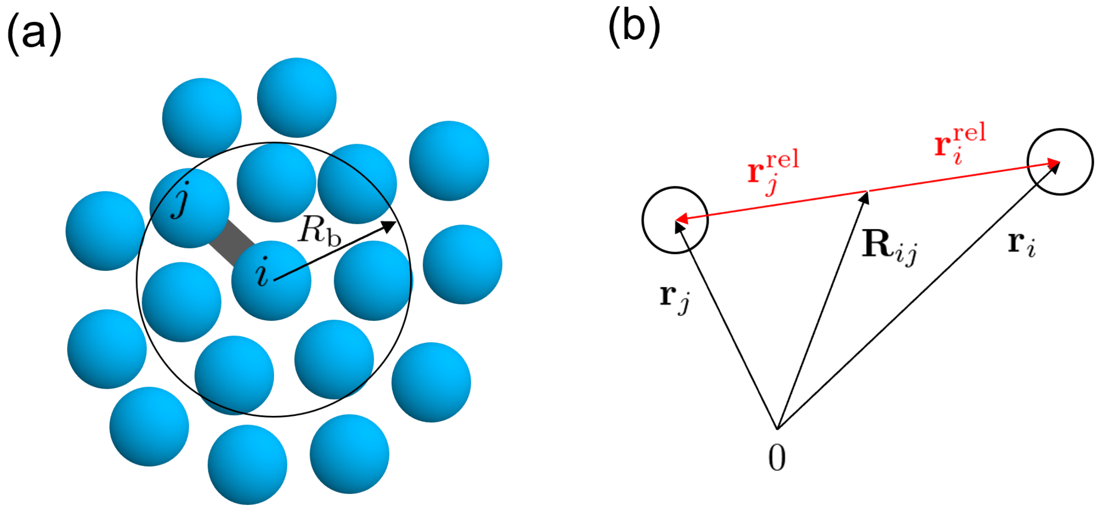

Supplementary Figure S2. **Schematic illustration of the construction of bonds.** (a): Making a bond connecting particles i and j. The sphere at the center of the particle i with the radius R_b defines its neighborhood. (b): The coordinates relative to the center of mass.

C. Constrained dynamics

We implement a constrained molecular dynamics simulation for the equilibrium dynamics and non-equilibrium heating-cooling process of the bonded system via the RATTLE method [4]. Since we are not aware of previous implementations of RATTLE with a simple Nosé-Hoover thermostat, we describe the details of our implementation below.

We recall the velocity-Verlet version of the Nosé-Hoover thermostat [5].

$$\mathbf{r}_i(t+\Delta t) = \mathbf{r}_i(t) + \mathbf{v}_i(t)\Delta t + \frac{(\Delta t)^2}{2}\left\{\mathbf{f}_i(t) - \zeta(t)\mathbf{v}_i(t)\right\}, \tag{13}$$

$$\mathbf{v}_i(t+\Delta t/2) = \mathbf{v}_i(t) + \frac{\Delta t}{2}\left\{\mathbf{f}_i(t) - \zeta(t)\mathbf{v}_i(t)\right\}, \tag{14}$$

$$\zeta(t+\Delta t) = \zeta(t) + \frac{\Delta t}{Q}\left[\sum_{i=1}^{N}\frac{1}{4}\left\{\mathbf{v}_i^2(t) + \mathbf{v}_i^2(t+\Delta t/2)\right\} - \frac{M+1}{2}T\right], \tag{15}$$

$$\mathbf{v}_i(t+\Delta t) = \frac{1}{1+\frac{\Delta t}{2}\zeta(t+\Delta t)}\left[\mathbf{v}_i(t+\Delta t/2) + \frac{\Delta t}{2}\mathbf{f}_i(t+\Delta t)\right], \tag{16}$$

where ζ is the degree of freedom of the thermostat and M is the number of degrees of freedom for the kinetic part. $M = 3N$ for the standard particle systems and $M = 3N_{\mathrm{m}} + 5N_{\mathrm{d}}$ for bonded systems in three dimensions. Q is a parameter that plays the role of a mass for the thermostat. We set $Q = 5$ and 100 for the system with $N = 1200$ and 32400, respectively. We set $\Delta t = 0.001$. The updates for the position and velocity can be combined in one equation, respectively:

$$\mathbf{r}_i(t+\Delta t) = \mathbf{r}_i(t) + \mathbf{v}_i(t)\Delta t + \frac{(\Delta t)^2}{2}\left\{\mathbf{f}_i(t) - \zeta(t)\mathbf{v}_i(t)\right\}, \tag{17}$$

$$\mathbf{v}_i(t+\Delta t) = \frac{1}{1+\frac{\Delta t}{2}\zeta(t+\Delta t)}\left[\mathbf{v}_i(t) + \frac{\Delta t}{2}\left\{\mathbf{f}_i(t) + \mathbf{f}_i(t+\Delta t) - \zeta(t)\mathbf{v}_i(t)\right\}\right]. \tag{18}$$

We now impose a rigid-body constraint between particles i and j. The position and velocity are constrained as follows:

$$\chi_{ij}(t) = \tilde{\mathbf{r}}_{ij}^2(t) - a_{ij}^2 = 0, \tag{19}$$

$$\frac{1}{2}\frac{\mathrm{d}\chi_{ij}(t)}{\mathrm{d}t} = \tilde{\mathbf{r}}_{ij}(t)\cdot\tilde{\mathbf{v}}_{ij}(t) = 0, \tag{20}$$

where $\tilde{\mathbf{r}}_{ij}(t) = \mathbf{r}_i(t) - \mathbf{r}_j(t)$, $\tilde{\mathbf{v}}_{ij}(t) = \mathbf{v}_i(t) - \mathbf{v}_j(t)$, and a_{ij} is the fixed bond length determined at $t = 0$, namely, $a_{ij} = |\mathbf{r}_i(t=0) - \mathbf{r}_j(t=0)|$. Below, we will also use the following notation, $\tilde{\mathbf{f}}_{ij}(t) = \mathbf{f}_i(t) - \mathbf{f}_j(t)$.

We add the constraint in Eq. (19) with the associated Lagrange multiplier λ_{ij} to the original potential U:

$$U \to U - \frac{\lambda_{ij}}{2}\chi_{ij}. \tag{21}$$

The extra term produces a force parallel to the relative position, as given by

$$-\nabla_i\left(-\frac{\lambda_{ij}}{2}\chi_{ij}\right) = \lambda_{ij}\tilde{\mathbf{r}}_{ij}. \tag{22}$$

We also add the constraint in Eq. (20) with the associated Lagrange multiplier μ_{ij}. After that, the update equations for the position and velocity become

$$\mathbf{r}_i(t+\Delta t) = \mathbf{r}_i(t) + \mathbf{v}_i(t)\Delta t + \frac{(\Delta t)^2}{2}\left\{\mathbf{f}_i(t) + \lambda_{ij}\tilde{\mathbf{r}}_{ij}(t) - \zeta(t)\mathbf{v}_i(t)\right\}, \tag{23}$$

$$\mathbf{v}_i(t+\Delta t) = \frac{1}{1+\frac{\Delta t}{2}\zeta(t+\Delta t)}\left[\mathbf{v}_i(t) + \frac{\Delta t}{2}\left\{\mathbf{f}_i(t) + \lambda_{ij}\tilde{\mathbf{r}}_{ij}(t) + \mathbf{f}_i(t+\Delta t) + \mu_{ij}\tilde{\mathbf{r}}_{ij}(t+\Delta t) - \zeta(t)\mathbf{v}_i(t)\right\}\right]. \tag{24}$$

The main task below is to determine λ_{ij} and μ_{ij} so that the constraint equations in Eqs. (19) and (20) always hold during the simulation.

We first determine λ_{ij} so that $\chi_{ij} = 0$ in Eq. (19) is satisfied at time t and $t + \Delta t$. With Eq. (23), we can write $\tilde{\mathbf{r}}_{ij}(t+\Delta t)$ as

$$\begin{aligned}\tilde{\mathbf{r}}_{ij}(t+\Delta t) &= \tilde{\mathbf{r}}_{ij}(t) + \tilde{\mathbf{v}}_{ij}(t)\Delta t + \frac{(\Delta t)^2}{2}\left\{\tilde{\mathbf{f}}_{ij}(t) + 2\lambda\tilde{\mathbf{r}}_{ij}(t) - \zeta(t)\tilde{\mathbf{v}}_{ij}(t)\right\} \\ &= A\,\tilde{\mathbf{r}}_{ij}(t) + B\,\tilde{\mathbf{v}}_{ij}(t) + C\,\tilde{\mathbf{f}}_{ij}(t),\end{aligned} \tag{25}$$

where $A = 1 + (\Delta t)^2\lambda$, $B = \Delta t - \frac{(\Delta t)^2}{2}\zeta(t)$, and $C = \frac{(\Delta t)^2}{2}$. Note that we used $\lambda = \lambda_{ij} = \lambda_{ji}$ due to the Newton's third law. Therefore, the constraint equation in Eq. (19) at $t + \Delta t$ becomes

$$\begin{aligned}\chi_{ij}(t+\Delta t) &= \tilde{\mathbf{r}}_{ij}^2(t+\Delta t) - a_{ij}^2 \\ &= (A\,\tilde{\mathbf{r}}_{ij}(t) + B\,\tilde{\mathbf{v}}_{ij}(t) + C\,\tilde{\mathbf{f}}_{ij}(t))^2 - a_{ij}^2 \\ &= a_{ij}^2A^2 + 2C(\tilde{\mathbf{r}}_{ij}(t)\cdot\tilde{\mathbf{f}}_{ij}(t))A + (B\tilde{\mathbf{v}}_{ij}(t) + C\tilde{\mathbf{f}}_{ij}(t))^2 - a_{ij}^2 = 0.\end{aligned} \tag{26}$$

We used $\tilde{\mathbf{r}}_{ij}^2(t) = a_{ij}^2$ and $\tilde{\mathbf{r}}_{ij}(t)\cdot\tilde{\mathbf{v}}_{ij}(t) = 0$. For diatomic dumbel systems, we can obtain λ analytically as follows.

$$\lambda = \frac{A-1}{(\Delta t)^2}, \tag{27}$$

$$A = \frac{-C(\tilde{\mathbf{r}}_{ij}(t)\cdot\tilde{\mathbf{f}}_{ij}(t)) + \sqrt{C^2(\tilde{\mathbf{r}}_{ij}(t)\cdot\tilde{\mathbf{f}}_{ij}(t))^2 - a_{ij}^2(B\tilde{\mathbf{v}}_{ij}(t) + C\tilde{\mathbf{f}}_{ij}(t))^2 + a_{ij}^4}}{a_{ij}^2}. \tag{28}$$

Next we determine μ_{ij} so that the second constraint equation in Eq. (20) is satisfied at time t and $t+\Delta t$. With Eq. (24), we can write $\tilde{\mathbf{v}}_{ij}(t+\Delta t)$ by

$$\begin{aligned}\tilde{\mathbf{v}}_{ij}(t+\Delta t) &= \frac{1}{D}\left[\tilde{\mathbf{v}}_{ij}(t) + \frac{\Delta t}{2}\left\{\tilde{\mathbf{f}}_{ij}(t) + 2\lambda\tilde{\mathbf{r}}_{ij}(t) + \tilde{\mathbf{f}}_{ij}(t+\Delta t) + 2\mu\tilde{\mathbf{r}}_{ij}(t+\Delta t) - \zeta(t)\tilde{\mathbf{v}}_{ij}(t)\right\}\right] \\ &= \frac{1}{D}\left[E\ \tilde{\mathbf{v}}_{ij}(t) + \frac{\Delta t}{2}\left\{\tilde{\mathbf{f}}_{ij}(t) + 2\lambda\tilde{\mathbf{r}}_{ij}(t) + \tilde{\mathbf{f}}_{ij}(t+\Delta t)\right\} + \mu\Delta t\tilde{\mathbf{r}}_{ij}(t+\Delta t)\right],\end{aligned}$$

where $D = 1 + \frac{\Delta t}{2}\zeta(t+\Delta t)$ and $E = 1 - \frac{\Delta t}{2}\zeta(t)$. We also used $\mu = \mu_{ij} = \mu_{ji}$. Thus, the second constraint equation in Eq. (20) becomes

$$\begin{aligned}\frac{1}{2}\frac{\mathrm{d}\chi_{ij}(t+\Delta t)}{\mathrm{d}t} &= \tilde{\mathbf{r}}_{ij}(t+\Delta t)\cdot\tilde{\mathbf{v}}_{ij}(t+\Delta t) \\ &= \frac{1}{D}\left[E(\tilde{\mathbf{r}}_{ij}(t+\Delta t)\cdot\tilde{\mathbf{v}}_{ij}(t)) + \frac{\Delta t}{2}\tilde{\mathbf{r}}_{ij}(t+\Delta t)\cdot\left\{\tilde{\mathbf{f}}_{ij}(t) + 2\lambda\tilde{\mathbf{r}}_{ij}(t) + \tilde{\mathbf{f}}_{ij}(t+\Delta t)\right\} + \mu\Delta t a_{ij}^2\right] \\ &= 0. \end{aligned} \tag{29}$$

We used $\tilde{\mathbf{r}}_{ij}^2(t+\Delta t) = a_{ij}^2$ to derive the above equation. Finally, we obtain the expression for μ as

$$\mu = -\frac{1}{a_{ij}^2\Delta t}\left[E(\tilde{\mathbf{r}}_{ij}(t+\Delta t)\cdot\tilde{\mathbf{v}}_{ij}(t)) + \frac{\Delta t}{2}\tilde{\mathbf{r}}_{ij}(t+\Delta t)\cdot\left\{\tilde{\mathbf{f}}_{ij}(t) + 2\lambda\tilde{\mathbf{r}}_{ij}(t) + \tilde{\mathbf{f}}_{ij}(t+\Delta t)\right\}\right]. \tag{30}$$

D. Initial velocities

The initial configuration of a randomly bonded glass former is already in thermal equilibrium, as we confirmed numerically. Yet, we have to assign the initial velocities so that the system follows the canonical ensemble at time $t = 0$. Under the presence of the rigid body bond constraints, we cannot use the standard expression for the Maxwell-Boltzmann distribution for each particle because it would break the constraint. Thus we pay special attention to initializing the velocities while keeping the rigid body constraints.

We assign the velocities for the monomers, the center of mass of dimers, and the relative movements of dimers, separately, based on the Hamiltonian H' in Eq. (8) at temperature $T = 1/\beta$. For the monomers, we can assign $\mathbf{v}_i(t=0) = \mathbf{v}_i^{\mathrm{MB}}$, where $\mathbf{v}_i^{\mathrm{MB}}$ is drawn by a Gaussian distribution with zero mean and variance T/m_i. For the center of mass of dimers, similarly, we can use $\mathbf{V}_{ij}(t=0) = \mathbf{V}_{ij}^{\mathrm{MB}}$ drawn by a Gaussian distribution with zero mean and variance T/M_{ij}. Special attention has to be paid to the relative motion. We consider the coordinates relative to the center of mass, $\mathbf{r}_i^{\mathrm{rel}} = \mathbf{r}_i - \mathbf{R}_{ij}$ and $\mathbf{r}_j^{\mathrm{rel}} = \mathbf{r}_j - \mathbf{R}_{ij}$, as shown schematically in Supplementary Figure S2b. $\mathbf{r}_i^{\mathrm{rel}}$ and $\mathbf{r}_j^{\mathrm{rel}}$ can be expressed by $\mathbf{r}_i^{\mathrm{rel}} = \frac{m_j}{M_{ij}}\tilde{\mathbf{r}}_{ij}$ and $\mathbf{r}_j^{\mathrm{rel}} = -\frac{m_i}{M_{ij}}\tilde{\mathbf{r}}_{ij}$, respectively. Considering both the center of mass and relative motion, we assign the velocities of particles i and j connected by a rigid bond as follows.

$$\mathbf{v}_i(t=0) = \mathbf{V}_{ij}(t=0) + \dot{\mathbf{r}}_i^{\mathrm{rel}}(t=0) = \mathbf{V}_{ij}^{\mathrm{MB}} + \frac{m_j}{M_{ij}}\dot{\tilde{\mathbf{r}}}_{ij}(t=0), \tag{31}$$

$$\mathbf{v}_j(t=0) = \mathbf{V}_{ij}(t=0) + \dot{\mathbf{r}}_j^{\mathrm{rel}}(t=0) = \mathbf{V}_{ij}^{\mathrm{MB}} - \frac{m_i}{M_{ij}}\dot{\tilde{\mathbf{r}}}_{ij}(t=0). \tag{32}$$

The remaining task is to assign $\dot{\tilde{\mathbf{r}}}_{ij}(t=0)$ in the above equations. Here we drop the indexes ij for simplicity. We express $\tilde{\mathbf{r}}$ by the spherical coordinate in $d=3$:

$$\tilde{\mathbf{r}} = \begin{cases} \tilde{x} = \tilde{r}\sin\theta\cos\varphi \\ \tilde{y} = \tilde{r}\sin\theta\sin\varphi \\ \tilde{z} = \tilde{r}\cos\theta \end{cases}$$

Since $\tilde{r}$ is constant, the relative velocity $\dot{\tilde{\mathbf{r}}}$ is given as follows.

$$\dot{\tilde{\mathbf{r}}} = \begin{cases} \dot{\tilde{x}} = \tilde{r}(\cos\theta\cos\varphi\ \dot{\theta} - \sin\theta\sin\varphi\ \dot{\varphi}) \\ \dot{\tilde{y}} = \tilde{r}(\cos\theta\sin\varphi\ \dot{\theta} + \sin\theta\cos\varphi\ \dot{\varphi}) \\ \dot{\tilde{z}} = -\tilde{r}\sin\theta\ \dot{\theta} \end{cases}$$

We thus initialize the values of $\dot{\theta}$ and $\dot{\varphi}$ to initialize $\dot{\mathbf{r}}$. Because the corresponding kinetic terms in Eq. (8) can be written by

$$-\beta\left(\frac{p_\theta^2}{2I}+\frac{p_\varphi^2}{2I\sin^2\theta}\right)=-\beta\left(\frac{I}{2}\dot{\theta}^2+\frac{I\sin^2\theta}{2}\dot{\varphi}^2\right), \tag{33}$$

we obtain the initial values of $\dot{\theta}$ and $\dot{\varphi}$ by the Gaussian distribution with zero mean and variance T/I and $T/(I\sin^2\theta)$, respectively.

E. Time correlation functions

To study the equilibrium dynamics, we compute the self part of the intermediate scattering functions for monomers, the center of mass of dimers, and all particles, given by

$$F_{\rm s}^{\rm Mono}(q,t)=\overline{\left\langle \frac{1}{N_{\rm m}}\sum_{i\in\mathcal{M}} e^{-i\mathbf{q}\cdot(\mathbf{r}_i(t)-\mathbf{r}_i(0))}\right\rangle_{\tilde{r}^{N_{\rm d}}}}, \tag{34}$$

$$F_{\rm s}^{\rm Di}(q,t)=\overline{\left\langle \frac{1}{N_{\rm d}}\sum_{\substack{i,j\in\mathcal{D}\\(i<j)}} e^{-i\mathbf{q}\cdot(\mathbf{R}_{ij}(t)-\mathbf{R}_{ij}(0))}\right\rangle_{\tilde{r}^{N_{\rm d}}}}, \tag{35}$$

$$F_{\rm s}^{\rm All}(q,t)=\overline{\left\langle \frac{1}{N}\sum_{i=1}^{N} e^{-i\mathbf{q}\cdot(\mathbf{r}_i(t)-\mathbf{r}_i(0))}\right\rangle_{\tilde{r}^{N_{\rm d}}}}, \tag{36}$$

respectively, where $\overline{\cdots}$ indicates an average over different bond realizations and $\langle\cdots\rangle_{\tilde{r}^{N_{\rm d}}}$ is a time average over a given realization $\tilde{r}^{N_{\rm d}}$. In practice we have averaged over 5–20 different realizations to calculate the time correlation functions. We set $q = 7.25$, the location of the main peak in the static structure factor [1].

In the main text we have shown the intermediate scattering functions obtained from the equilibrium dynamics at $T = 0.6$ and $c = 0.95$, computed from the positions of monomers, $F_{\rm s}^{\rm Mono}(q,t)$, the center of mass of the dimers, $F_{\rm s}^{\rm Di}(q,t)$, and all particles, $F_{\rm s}^{\rm All}(q,t)$, together with the corresponding mean-squared displacements and rotational correlation function (for dimer molecules). $F_{\rm s}^{\rm Di}(q,t)$ displays a higher value of the plateau than the one found in $F_{\rm s}^{\rm Mono}(q,t)$ or $F_{\rm s}^{\rm All}(q,t)$. However, these functions relax on essentially the same timescale which is evidence that the monomers and dimers have a quite similar relaxation dynamics. We define the relaxation time τ_α as the time at which the intermediate scattering function decays to $1/e$ and present the τ_α vs. $1/T$ plot for $c = 0.5$ in Supplementary

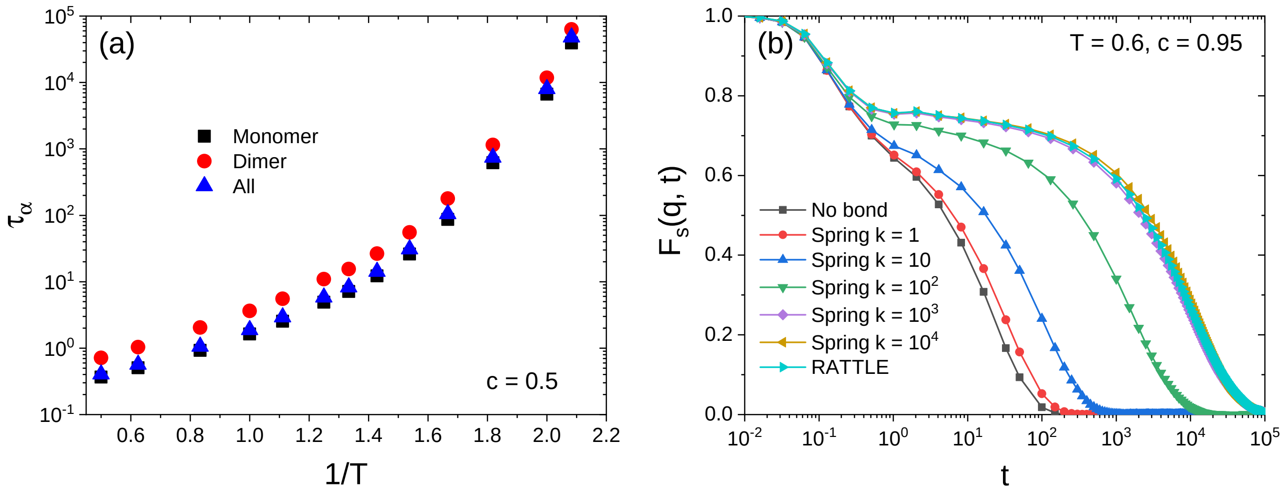

Supplementary Figure S3. **Equilibrium dynamics for bonded systems.** (a): Relaxation time τ_α vs. the inverse of temperature $1/T$ for $c = 0.5$. (b): Simulations of harmonic spring method with several spring constant k. $F_{\rm s}(q,t)$ from all particles for $T = 0.6$ and $c = 0.95$. The system size is $N = 1200$.

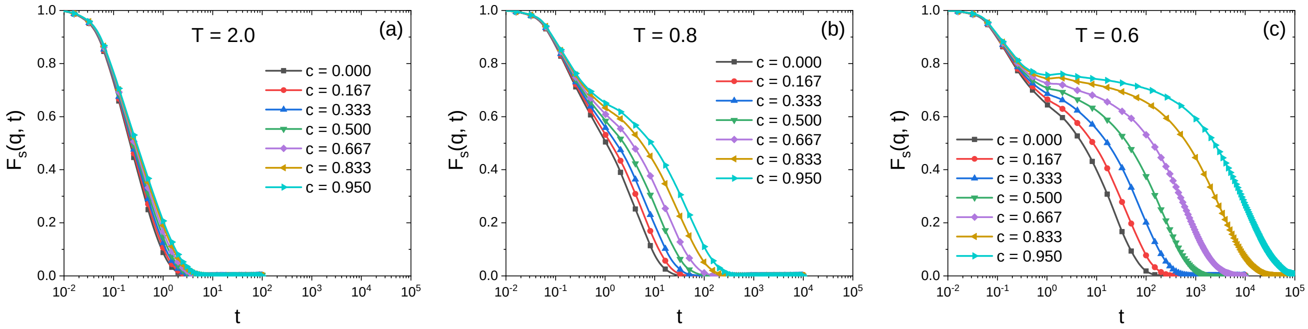

Supplementary Figure S4. **Self intermediate scattering function $F_{\rm s}(q,t)$ for all particles for several c.** (a): $T = 2.0$. (b): $T = 0.8$. (c): $T = 0.6$. The system size is $N = 1200$.

Figure S3a. The relaxation time τ_α for the center of mass of dimers exceeds τ_α for the monomers by a factor of two, independent of T, which shows that the dynamics of the two types of particles does not decouple from each other, and we have checked that this is also the case for the other values of c. We conclude that the three definitions of the intermediate scattering functions provide us with essentially the same information in terms of structural relaxation. We thus decided to stick to $F_{\rm s}^{\rm All}(q,t)$ for most of our results, and drop the superscript unless otherwise specified.

In the main text, we have also presented a rotational correlation function for dimers, $C(t)$, defined by

$$C(t) = \overline{\left\langle \frac{1}{2N_{\rm d}} \sum_{i,j\in\mathcal{D}} \mathbf{n}_{ij}(t)\cdot\mathbf{n}_{ij}(0) \right\rangle}_{\tilde{r}^{N_{\rm d}}}, \tag{37}$$

where $\mathbf{n}_{ij} = \tilde{\mathbf{r}}_{ij}/a_{ij}$ is the unit vector of the orientation of a dumbbell molecule composed of particles i and j. $C(t)$ is an analog of the dynamical correlation function measured in dielectric experiments [6].

In order to check our implementation of RATTLE, we perform independent molecular simulations by replacing the rigid bonds by a harmonic springs. These harmonic springs are introduced in addition to the original potential energy, namely,

$$U \to U + k \sum_{\substack{i,j\in\mathcal{D}\\(i<j)}} (\tilde{r}_{ij} - a_{ij})^2, \tag{38}$$

where k is the spring constant. Hence $k \to \infty$ should correspond to the rigid body constraint. Supplementary Figure S3b shows $F_{\rm s}(q,t)$ for $T = 0.6$ and $c = 0.95$ for different values of k together with the results from the simulations using RATTLE. We find that simulations with $k = 10^3 - 10^4$ converge to the results from the simulations using RATTLE, which is evidence that our algorithm to constrain the bonds is correct.

We present $F_{\rm s}(q,t)$ for different T and c in Supplementary Figure S4 to complement the results shown in the main text. We find that the effect of bonding is very small if temperature is high, $T = 2.0$ in Supplementary Figure S4a. Yet the effect is enhanced significantly as T is decreased, akin to the results for randomly pinned systems [7, 8]. In order to see the influence of T and c together, we have shown the iso-τ_α curves in the T versus c plane in the main text. The iso-τ_α curves increase with increasing c, which is again similar to the results found for randomly pinned systems [9, 10].

Finally we present a comparison between the self and collective parts of the intermediate scattering functions. It has been reported that the collective part shows apparent freezing in randomly pinned fluids, which makes the analysis difficult [10]. In Supplementary Figure S5a we show the self and collective parts for the randomly-bonded glass formers at $T = 0.6$ for different values of c. One recognizes that, in contrast to the pinned systems, the collective part also relaxes to zero and that the relaxation time is slightly larger than the one for the self part. Interestingly, however, the ratio between the two timescales is about a factor two irrespectively of c, as shown in Supplementary Figure S5b, i.e., self and collective correlators do not decouple. Therefore we can conclude that the self part gives reliable dynamical information about the system.

F. Kinetic and mechanical stability

In Supplementary Figure S6 we show additional supporting data for the heating/cooling process for the original (non-bonded) system ($c = 0$) (a) and bonded system ($c = 0.95$) (b), with two distinct values of the heating/cooling

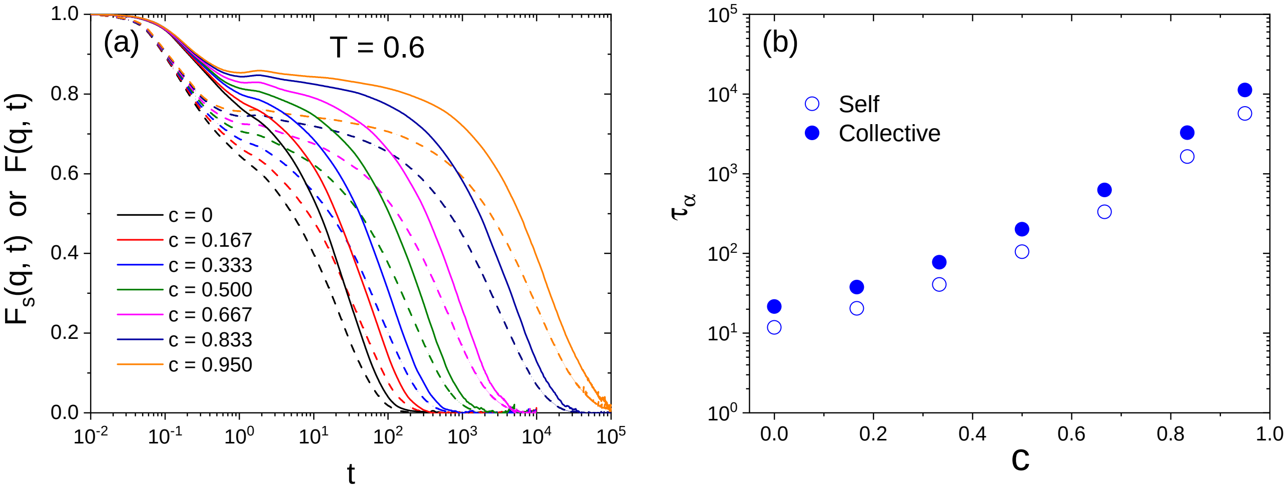

Supplementary Figure S5. **Comparison between the self and collective parts of the intermediate scattering function.** (a): Self (dashed curves) and collective (solid curves) intermediate scattering functions at $T = 0.6$ for different c. (b): Relaxation time τ_α vs. c, computed from the data in (a). The system size is $N = 1200$.

rate K. In both cases, we start from the system prepared at $T = 0.42$, and we heat it up to $T = 2.0$ (the first heating in the main text) and cool it back down to $T = 0.42$. We do not report the second heating here. The data for the non-bonded system of monomers ($c = 0$, Supplementary Figure S6a) do not show a marked hysteresis, as expected for an ordinary glass. On the contrary, the data for the bonded system ($c = 0.95$, Supplementary Figure S6b) show a marked and strongly K-dependent hysteresis, characteristic of an ultrastable glass.

We also report additional data for our mechanical tests. We firstly prepare an equilibrium configuration of a randomly bonded glass at temperature T and concentration c. We then perform a rapid quench down to zero temperature by using the conjugate gradient method [11]. To this end, we replace the rigid bond constraint with the harmonic spring in Eq. (38) with sufficiently large k. We vary k from 10^1 to 10^4 and find that simulations above $k = 10^3$ converge, which is consistent with the equilibrium dynamics in Supplementary Figure S3b. Once we prepared a zero-temperature glass sample, we perform an athermal quasi-static shear simulation using Lees-Edwards boundary conditions [12], still keeping a harmonic spring constraint. This method consists of a succession of tiny uniform shear deformations with $\Delta\gamma = 10^{-4}$ followed by energy minimization via the conjugate-gradient method.

We compute the shear stress σ by the Irvine-Kirkwood formula [13] using the potential U in Eq. (38). In principle

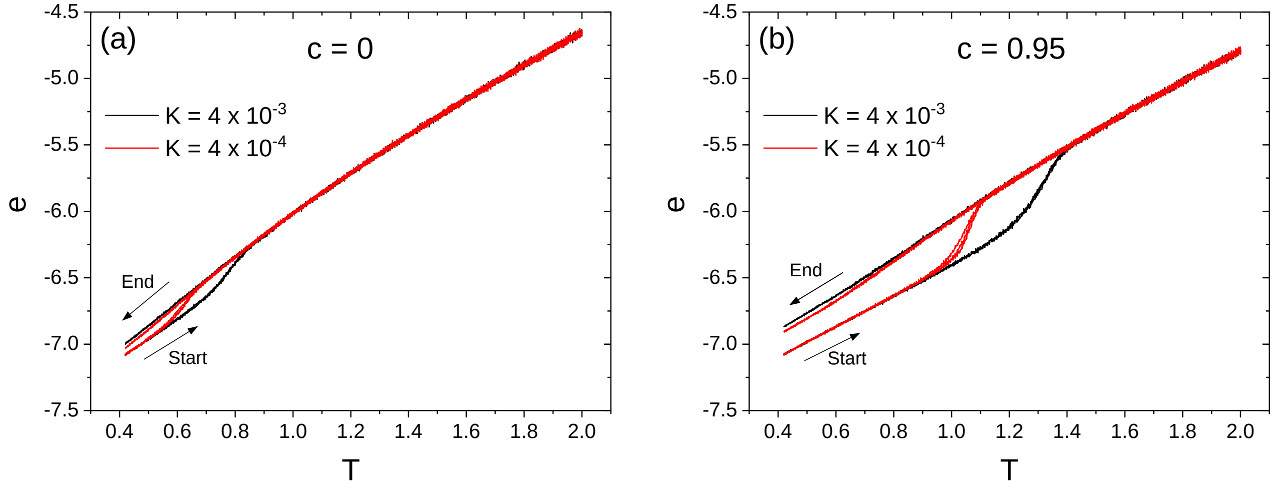

Supplementary Figure S6. **Potential energy per particle e as a function of temperature T for the heating-cooling process.** (a): The original system without bond ($c = 0$). (b): The bonded system ($c = 0.95$). Three independent realizations are presented. The system size is $N = 32400$.

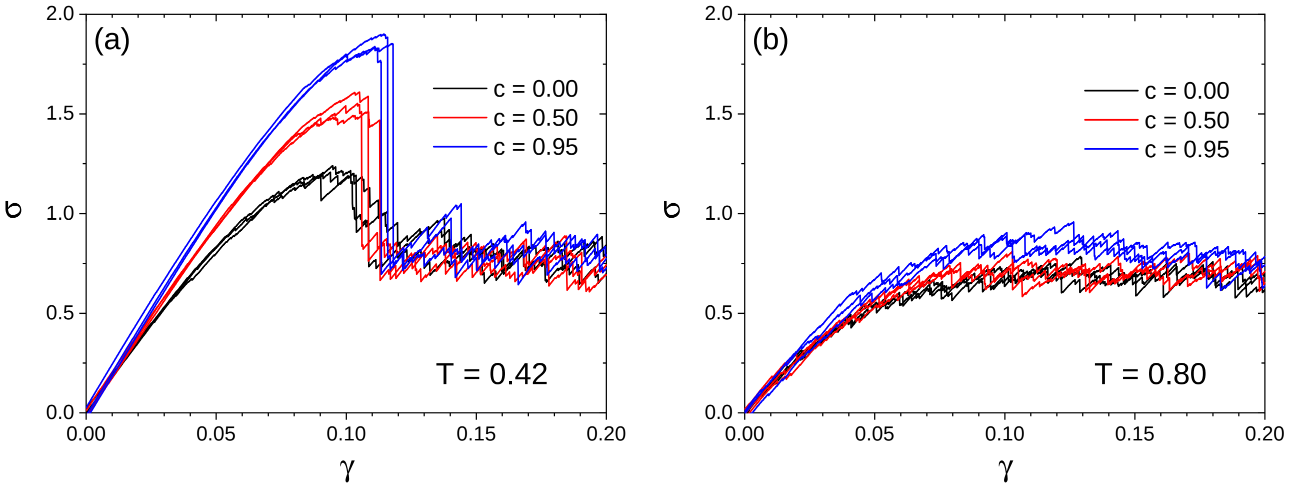

Supplementary Figure S7. **Shear stress σ versus strain γ curves for randomly bonded glass formers generated for $c = 0$, 0.5, and 0.95 at the preparation temperature T.** (a): $T = 0.42$. (b): $T = 0.8$. Three individual realizations are shown. The system size is $N = 32400$.

the contribution from the spring term should be strictly zero in the perfectly rigid-bond simulations. However, in practice it produces a finite value of the shear stress in our harmonic spring simulations, which we neglect in the following since this additional contribution does not change the conclusions of this paper.

We present the stress vs. strain curves for $c = 0$, 0.5, and 0.95, prepared at $T = 0.42$ and $T = 0.8$ in Supplementary Figure S7. Whereas the $T = 0.42$ data show sharp brittle yielding for $c = 0.5$ and 0.95, the $T = 0.8$ data do not show this sharp yielding. This means that the sharp brittle yielding for $T = 0.42$ originates from the fact that the system is sitting deep inside the glassy landscape, and does not originate from the bonding process itself.

Supplementary Note 3. BONDING IN PATCHY COLLOID EXPERIMENT

In this section, we describe the experimental method and the results obtained for bonded colloidal particles dispersed in a quasi-two-dimensional sample. In the experiment, we use the concentration of salt to induce an attraction between the patchy colloidal particles that is sufficiently strong to create bonds between hydrophobic patches [14].

The patchy particles were produced as follows [14–16]: First, a 5-nm-thick chromium layer followed by a 40-nm-thick gold layer was thermally deposited onto the hemispheres of spherical silica particles (2.7 μm diameter, polydispersity 2.5%, HIPRESICA TS, UEXC). The patches were chemically etched as described in Ref. [15], and their size estimated from our etching-time dependence experiment is $\theta_{\rm ap} \sim 50°$, the opening angle of a circular patch measured from its center. The patches were hydrophobized by 1-octadecanethiol (90%, Sigma-Aldrich) [14].

The produced one-patch particles were mixed with spherical silica particles (2.0 μm $\pm$2.5% in diameter, HIPRESICA TS, UEXC) in water (18.2MΩ), and the particles were dispersed by ultrasonication for 1 minute. The dispersion of the binary particle mixture was confined in the lower layer of the experimental cell (Supplementary Figure S8). The top and bottom plates are glass slides hydrophilized by plasma cleaning. The upper part of the cell, which was separated from the lower part by a semipermeable membrane, had an inlet and outlet channel to exchange the liquid in the

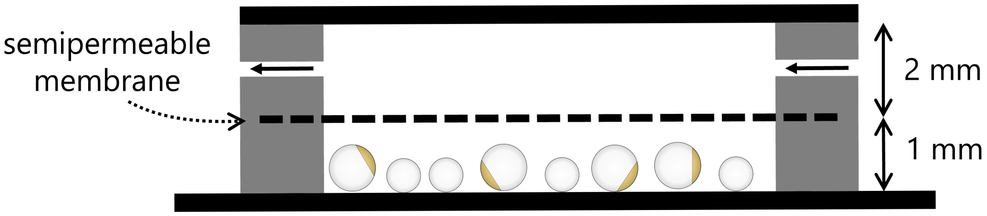

Supplementary Figure S8. **Sketch of the experimental cell.**

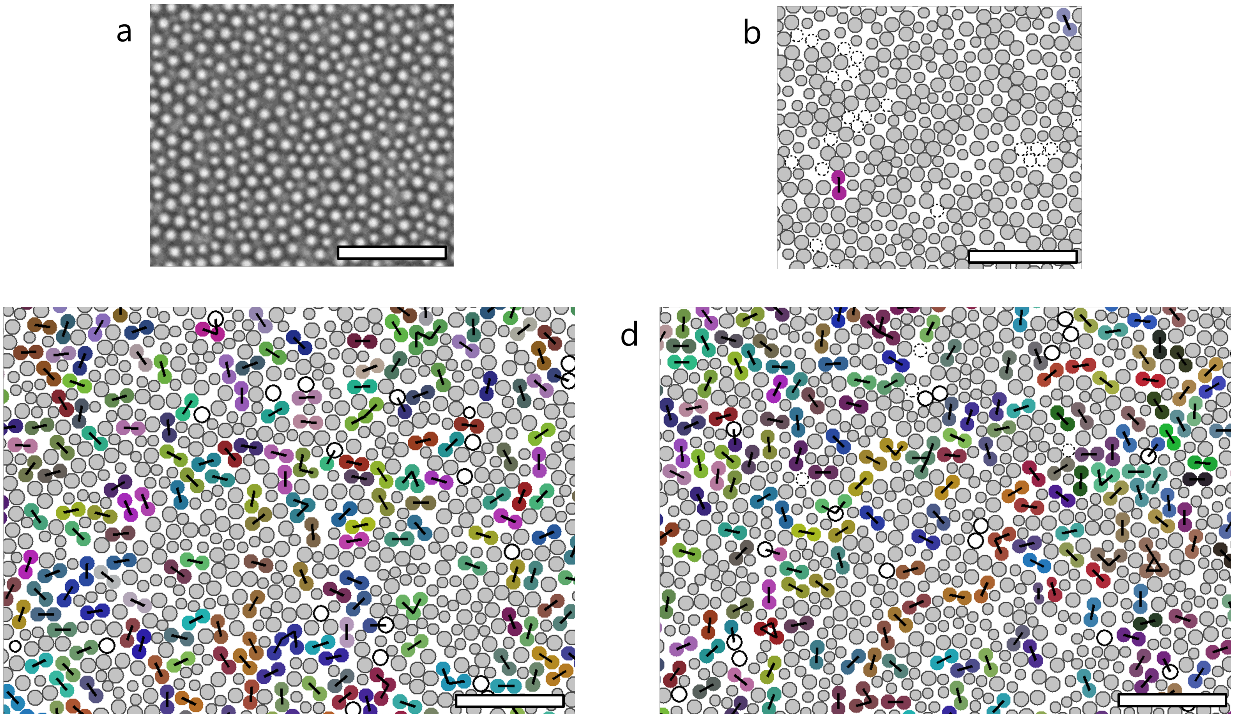

Supplementary Figure S9. **Two-dimensional dispersions of patchy and non-patchy particle mixture.** (a): A microscopy image at 0.0 mM. Large particles are patchy and small particles are without patch. 1 % of particles were originally bonded, survived the initial sonication. $\varphi = 0.60$. (b-d): Reconstructed images from the analysis on particle motion. The classification is described in the text. The salt concentration and area fraction are 0.0 mM and $\varphi = 0.63$ for (b), 4.0 mM and $\varphi = 0.63$ for (c) and 6.0 mM and $\varphi = 0.62$ for (d). Scale bars: 20 μm.

upper layer. Thus, by changing the NaCl concentration in the upper layer, the salt concentration in the lower layer can be tuned by the molecular exchange *via* the semipermeable membrane without inducing convection in the lower layer. We increased the NaCl concentration in the lower layer stepwise from 0.0, 1.0, 2.0, 4.0, 6.0, 8.0 to 10.0 mM, where the salt solution in the upper layer was exchanged several times in more than an hour for each concentration step. The particle dispersion was equilibrated for more than two hours after each concentration step. The two-dimensional dispersion of the sedimented particles was observed with optical microscopy (IX73 with an objective LUCPlanFLN 40X/0.60, Olympus) after the equilibration, exhibiting no further development of dispersed state such as bonding between particles or sticking of particles to the bottom surface, obtained by the analyses described below. The microscopy images were taken at a rate of 2 frames per second. A patch on a 2.7 μm particle is thin, and thus it is almost invisible in the images, see Supplementary Figure S9a. Particle size and position were obtained from the binarized images, and the trajectory of each particle was determined from this data.

Supplementary Figure S9 shows a microscopy image and reconstructed images containing the information of the dispersed state obtained from 100 successive images. In the experiment, the particle area fraction in the two-dimensional plane was $\varphi \simeq 0.6$, the number ratio of the patchy to non-patchy particle was 7:3, and the number of analyzed particles in an image was ≈ 2400. In the reconstructed images we classified the dispersed state of a particle into 4 types. When the variance of the distance between a particle and its neighbor is less than 0.1 μm^2, it is regarded as bonded (filled color circle with solid black lines denoting bonding). When the variance of the position of a particle is less than 0.03 μm^2, it is regarded as immobile. (Because of the noise in the images, the position of a particle and distance between aggregating particles obtained from the image processing fluctuate even when they were actually constant.) The other particles are classified as monomers (filled gray circle), except for the particles that failed to be tracked (dotted open circle). A change in the so-defined type of a given particle was rarely observed under these experimental conditions: No change was found at 0.0 and 6.0 mM even in an hour observation, and one or two bond breaks were typically found at 4.0 mM in an hour, corresponding to ~ 0.3 % of all bonds.

We found that a fairly large amount of patchy particles were bonded while being dispersed at 4.0 and 6.0 mM as shown in Supplementary Figures S9c and S9d. In the figure most of the bonded particles are large, i.e. patchy, strongly suggesting that hydrophobic patches were bonded [14]. The number fractions of bonded particles were 1.4,

39 and 36 % for 0.0, 4.0 and 6.0 mM, respectively, corresponding to bonding of $\sim$ 60 % of patchy particles at 4.0 and 6.0 mM. In addition, the weight fractions of dimers in all the bonded particles were 90 and 91% for 4.0 and 6.0 mM, i.e., most of the bonded particles formed dimers. At these concentrations, the fraction of immobile particles were less than 1 %.

Further increase in salt concentration increased the fraction of bonded particles to some extent, that reached 48 and 63 % at 8.0 and 10.0 mM, respectively, although immobile particles increased more rapidly, 3.1 and 7.3 % at the two concentrations. The weight fractions of dimers in all the bonded particles decreased, 75 and 63 % at 8.0 and 10.0 mM, respectively, indicating that large aggregates increased more than dimers. The bonding and immobilization of the small, non-patchy particles also increased in the aggregates and immobile particles at these concentrations, suggesting the increase of bonding between patch-silica, silica-silica and silica-glass surfaces. Note that the multi-bonding configurations allowed for the patch size would also contribute to the formation of the large aggregates.

In conclusion, this preliminary experiment demonstrates that colloidal particles with a hydrophobized patch can be bonded at any time in a dense dispersion by changing salt concentration, where $\sim$ 4.0 to 6.0 mM would be the best for achieving both bonding and dispersity of particles.

Supplementary Note 4. SUPPLEMENTARY REFERENCES

[1] Walter Kob and Hans C Andersen, "Testing mode-coupling theory for a supercooled binary Lennard-Jones mixture I: The van Hove correlation function," Phys. Rev. E **51**, 4626 (1995).

[2] Peter Scheidler, Walter Kob, and Kurt Binder, "The relaxation dynamics of a supercooled liquid confined by rough walls," J. Phys. Chem **108**, 6673–6686 (2004).

[3] Steve Plimpton, Paul Crozier, and Aidan Thompson, "Lammps-large-scale atomic/molecular massively parallel simulator," Sandia National Laboratories **18**, 43 (2007).

[4] Hans C Andersen, "Rattle: A "velocity" version of the shake algorithm for molecular dynamics calculations," J. Comput. Phys. **52**, 24–34 (1983).

[5] Daan Frenkel and Berend Smit, *Understanding molecular simulation: from algorithms to applications*, Vol. 1 (Elsevier, 2001).

[6] Mark D Ediger, C Austen Angell, and Sidney R Nagel, "Supercooled liquids and glasses," The journal of physical chemistry **100**, 13200–13212 (1996).

[7] Robert L Jack and Christopher J Fullerton, "Dynamical correlations in a glass former with randomly pinned particles," Phys. Rev. E **88**, 042304 (2013).

[8] Saurish Chakrabarty, Rajsekhar Das, Smarajit Karmakar, and Chandan Dasgupta, "Understanding the dynamics of glass-forming liquids with random pinning within the random first order transition theory," J. Chem. Phys. **145**, 034507 (2016).

[9] Walter Kob and Ludovic Berthier, "Probing a liquid to glass transition in equilibrium," Phys. Rev. Lett. **110**, 245702 (2013).

[10] Misaki Ozawa, Walter Kob, Atsushi Ikeda, and Kunimasa Miyazaki, "Equilibrium phase diagram of a randomly pinned glass-former," PNAS **112**, 6914–6919 (2015).

[11] Stephen Wright and Jorge Nocedal, "Numerical optimization," Springer Science **35**, 7 (1999).

[12] Craig E Maloney and Anael Lemaitre, "Amorphous systems in athermal, quasistatic shear," Phys. Rev. E **74**, 016118 (2006).

[13] Michael P Allen and Dominic J Tildesley, *Computer simulation of liquids* (Oxford university press, 2017).

[14] Qian Chen, Sung Chul Bae, and Steve Granick, "Directed self-assembly of a colloidal kagome lattice," Nature **469**, 381–384 (2011).

[15] Qian Chen, Erich Diesel, Jonathan K Whitmer, Sung Chul Bae, Erik Luijten, and Steve Granick, "Triblock colloids for directed self-assembly," J. Am. Chem. Soc. **133**, 7725–7727 (2011).

[16] Yasutaka Iwashita and Yasuyuki Kimura, "Orientational order of one-patch colloidal particles in two dimensions," Soft Matter **10**, 7170–7181 (2014).
